# Risk factors for adverse events associated with endoscopic retrograde cholangiopancreatography in patients with surgically altered anatomy: a retrospective study

**DOI:** 10.1186/s12876-021-02031-w

**Published:** 2021-11-27

**Authors:** Xiaojia Chen, Fan Wang, Jing Liu, Wenhui Tao, Zhang Zhang, Tingting Cao, Jun Fang, Qiu Zhao

**Affiliations:** 1 Department of Gastroenterology, Zhongnan Hospital of Wuhan University, No. 169, Donghu Road, Wuchang District, Wuhan, 430071 Hubei Province China; 2 Hubei Clinical Center and Key Lab of Intestinal and Colorectal Diseases, Wuhan, China; 3 Emergency Center, Zhongnan Hospital of Wuhan University, Wuhan, China

**Keywords:** Surgically altered anatomy, ERCP, Adverse events, Risk factors

## Abstract

**Introduction:**

Endoscopic retrograde cholangiopancreatography (ERCP) is considered to be a challenge in patients with surgically altered anatomy. We aimed to identify the risk factors of ERCP-related adverse events in patients with surgically altered anatomy in our center.

**Methods:**

We included patients with surgically altered anatomy who underwent ERCP between April 2017 and December 2020 at our center. Clinical characteristics and outcomes were analyzed in univariate and multivariate methods to identify the risk factors for adverse events.

**Results:**

A total of 121 ERCP procedures were performed in 93 patients. The papilla or surgical anastomosis was successfully reached in 113 cases (93.4%). Diagnostic success was achieved in 106 cases (93.8%) and subsequent therapeutic success was achieved in 102 cases (96.2%). ERCP-related adverse events occurred in 31 cases (25.6%). In univariate analysis, not first time ERCP attempt, a CBD stone diameter ≥ 15 mm, multiple cannulation attempts, endoscopic papillary balloon dilation, endoscopic papillary large balloon dilation, endoscopic retrograde biliary drainage, biopsy in the bile duct or papilla, mechanical lithotripsy use, and stone retrieval basket were associated with ERCP-related adverse events. In multivariate analysis, multiple cannulation attempts (OR 5.283; 95% CI 1.088–25.659; *p* = 0.039), endoscopic papillary balloon dilation (OR 4.381; 95% CI 1.191–16.114; *p* = 0.026), and biopsy in the bile duct or papilla (OR 35.432; 95% CI 2.693–466.104; *p* = 0.007) were independently associated with ERCP-related adverse events.

**Conclusions:**

ERCP in patients with surgically altered anatomy was feasible and safe. Interventions including multiple cannulation attempts, endoscopic papillary balloon dilation, and biopsy in the bile duct or papilla were independent risk factors for ERCP-related adverse events.

## Introduction

Since its first description in 1968, endoscopic retrograde cholangiopancreatography (ERCP) has been universally applied in the diagnosis and treatment of pancreaticobiliary diseases [1–3]. The success rate of ERCP in patients with normal anatomy was estimated to be 95%, conducted by experienced endoscopists [4]. However, it is considered a challenge to complete ERCP successfully in patients with surgically altered anatomy. This may due to an increase in the intestine length and sharp angulation of the afferent limb [5, 6]. Alternative methods such as percutaneous or surgical interventions will be selected for rescue therapy, which brings about more invasiveness and complications [7].

Reconstruction methods of surgically altered anatomy include Billroth-I and Billroth-II gastrectomy, gastrectomy and hepaticojejunostomy with Roux-en-Y, and pancreaticoduodenectomy. The three major challenges during the ERCP procedure are reaching the native papilla or biliopancreatoenteric anastomosis, cannulating the bile or pancreatic duct, and performing relevant interventions [8, 9]. It is incredibly important to thoroughly understand a patient’s anatomy, which helps to guide the endoscopic access and therapy [10]. Considering the complexity of surgically altered anatomy, extensive experience is required. Many studies have focused on different reconstruction methods [11–13], yet only a single method was discussed in most of them.

Several types of enteroscopes have been used clinically, including forward-viewing endoscopes and side-viewing endoscopes. Device-assisted enteroscopes (DAEs) were recently introduced to increase the success rate of ERCP in patients with surgically altered anatomy, such as double-balloon enteroscopes, single-balloon enteroscopes and spiral enteroscopes. The success rate was reported to be close to 75% with DAEs, while at most 51% with conventional equipment [14]. However, success rates varied in existing studies [15–20], and new complications occurred subsequently due to the availability of new approaches [21]. No standardized strategy has been established to choose the most effective and safe approach.

To accurately evaluate the clinical appropriateness of ERCP, it is essential to understand potential adverse events of the procedure. ERCP in patients with surgically altered anatomy is considered more challenging and leads to a higher risk of adverse events. Specific attention has been paid to perforations owing to the complexity of anatomy [22]. Risk factors of ERCP-related complications for normal anatomy have been reported before [23–26], but few focused on surgically altered anatomy.

Patients with surgically altered anatomy are more susceptible to biliopancreatic complications, necessitating further ERCP interventions [27, 28]. In this study, we aimed to identify the risk factors of ERCP-related adverse events in patients with surgically altered anatomy in our center.

## Methods

### Patients

Patients with altered upper gastrointestinal anatomy who underwent ERCP between April 2017 and December 2020 at our center were identified from our hospital’s medical records and endoscopic database. The reconstruction method included Billroth-I gastrectomy, Billroth-II gastrectomy, Roux-en-Y gastrectomy, Hepaticojejunostomy with Roux-en-Y, and Pancreaticoduodenectomy.

The following patients’ information was recorded: age, gender, reconstruction method and its occasion, reasons for ERCP, and type of intervention. The diameter and number of the common bile duct (CBD) were assessed. Furthermore, the type of endoscope used during the procedure was recorded for detailed analysis. This study was approved by the institutional review board of our hospital.

### ERCP procedure

ERCP was performed under conscious sedation using intravenous butorphanol and diazepam. The prone position or the left lateral position was chosen at the beginning. The procedure was performed by 2 experienced endoscopists (HW and QZ) who had completed endoscopy training and performed more than 500 ERCPs every year. Written informed consent for ERCP had been achieved from all patients. A gastroscope (GIF HQ290, Olympus, Tokyo, Japan), duodenoscope (JF 260V, Olympus), standard colonoscope (CF HQ290L/I, Olympus), long colonoscope (CF HQ290L/I, Olympus), or short-type single-balloon enteroscope (SIF Q260, Olympus) was used. A transparent hood (D-201, Olympus) was attached to the tip of the endoscope to improve the visualization.

**Fig. 1:**
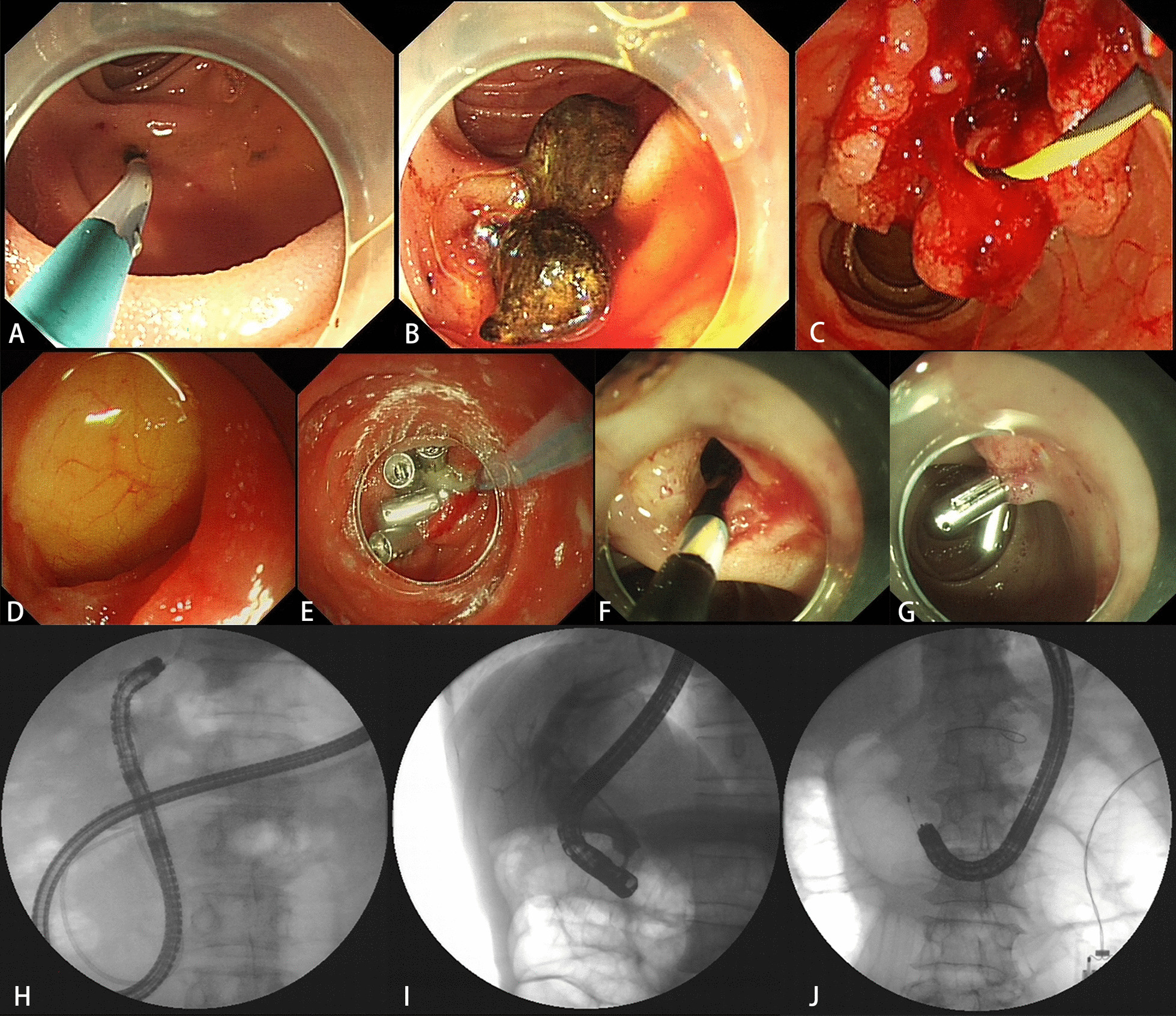
ERCP procedure. **A** A surgical anastomosis was reached in a patient who had underwent pancreaticoduodenectomy. **B** Common bile duct stones were detected. **C** Biopsy was performed in a case of bile duct cancer. **D** A perforation occurred in the acute angulation of the afferent loop. **E** The perforation was treated with purse string suture endoscopically. **F** A case of delayed bleeding occurred. **G** The delayed bleeding was treated with emergency endoscopic hemostasis using balloon compression and hemostatic clips. **H** A short-type single-balloon enteroscope was used. **I** A duodenoscope was used. **J** A standard colonoscope was used

When reaching the native papilla or the surgical anastomosis, selective biliary cannulation was performed. Papilla pre-cut was performed in cases of difficult cannulation. Endoscopic papillary balloon dilation (EPBD) was performed using a balloon dilator. For large common bile duct stones, mechanical lithotripsy was operated. Stone removal was performed via balloon catheter or retrieval basket. Biopsy was performed in cases of bile duct cancer. Concerning malignant biliary obstruction, a metal stent was inserted with a 7F delivery system. If it failed to reach the native papilla or the surgical anastomosis, another ERCP would be attempted after several days. The detailed ERCP procedure was shown in Fig. 1.

### Definitions

Technical success was defined as reaching the papilla or surgical anastomosis successfully. Diagnostic success was defined as success in selective cannulation and cholangiography. Therapeutic success was defined as the achievement of the expected therapeutic goal (e.g., endobiliary biopsy, stone removal and stent placement). Multiple cannulation attempts were defined as the cannulation of more than one attempt during the procedure.

ERCP-related adverse events included pancreatitis, hyperamylasemia, perforation, bleeding, cholangitis and mucosal laceration [29]. Post-ERCP pancreatitis was defined as typical abdominal pain accompanied by serum pancreatic enzyme (amylase or lipase) levels elevated by three times the upper limit of normal for more than 24 h after the procedure. Hyperamylasemia was defined as an elevation of serum pancreatic enzyme levels without typical abdominal pain. Perforation was defined as air or luminal contents outside the gastrointestinal tract. Bleeding was defined as hematemesis and/or melena or a decrease in hemoglobin of more than 2 g/dL. Cholangitis was defined as a fever of more than 38 °C sustaining at least 24 h with cholestasis. Mucosal laceration was defined as destruction of completeness of the mucosal during the ERCP procedure.

### Statistical analysis

Categorical variables were compared with chi-square or Fisher exact test, while continuous variables were analyzed with Student t-test. To identify potential risk factors for ERCP-related adverse events, univariate logistic regression analysis with a backward likelihood ratio was performed. For variables with a *P* value less than 0.2 in the univariate analysis, a forward stepwise multivariate logistic regression analysis was carried out to find the independent risk factors. A *P* value of less than 0.05 was considered statistically significant. Odds ratios (ORs) with 95% confidence intervals (CIs) were calculated. All the statistical analyses were performed with IBM SPSS Statistics software, version 26.0 (IBM Corp., Armonk, NY).

## Results

### Patient characteristics

A total of 121 ERCP procedures were performed in 93 patients with surgically altered anatomy between April 2017 and December 2020 in our center. The mean age of the 93 patients was 66.3 ± 12.0 years, among them 68 patients (73.1%) were male. 27 patients (29.0%) had a history of prior cholecystectomy. Reconstruction methods and indications for ERCP were also provided in Table 1. Billroth-II gastrectomy (71.0%) was superior in numbers. The main indication for ERCP was choledocholithiasis (61.2%), followed by malignant biliary obstruction (19.0%). 78 patients (83.9%) had a native papilla and 14 patients (15.1%) had a diverticulum.

**Table 1:** Baseline characteristics

Patients, n	93
Age, mean (SD), years	66.3 (± 12.0)
Sex (male/female), n	68/25
Prior cholecystectomy, n (%)	27 (29.0)
*Reconstruction method, n (%)*	
Billroth-I gastrectomy	8 (8.6)
Billroth-II gastrectomy	66 (71.0)
Roux-en-Y gastrectomy	3 (3.2)
Hepaticojejunostomy with Roux-en-Y	3 (3.2)
Pancreaticoduodenectomy	13 (14.0)
*Indications for ERCP, n (%)*	
Choledocholithiasis	74 (61.2)
Hepatolithiasis	10 (8.3)
Pancreatolithiasis	4 (3.3)
Benign biliary obstruction	5 (4.1)
Malignant biliary obstruction	23(19.0)
Anastomotic stenosis	5 (4.1)
Presence of diverticulum, n (%)	14 (15.1)
Presence of native papilla, n (%)	78 (83.9)
With a transparent hood, n (%)	78 (64.5)

ERCP: Endoscopic retrograde cholangiopancreatography

### Clinical outcomes

ERCP was performed with a gastroscope, duodenoscope, standard colonoscope, long colonoscope or short-type single-balloon enteroscope (SBE) (Table 2). Gastroscope (47.1%) was the most common choice, followed by duodenoscope (25.6%).

**Table 2:** Type of endoscope, n (%)

	Gastroscope	Duodenoscope	Standard colonoscope	Long colonoscope	Short-type single-balloon enteroscope	Total
Billroth-I gastrectomy	0	8 (100)	0	0	0	8
Billroth-II gastrectomy	52 (62.7)	23 (27.8)	6 (7.2)	1 (1.2)	1 (1.2)	83
Roux-en-Y gastrectomy	0	0	2 (50)	0	2 (50)	4
Hepaticojejunostomy with Roux-en-Y	2 (28.6)	0	0	0	5 (71.4)	7
Pancreaticoduodenectomy	3 (15.8)	0	4 (21.1)	7 (36.8)	5 (26.3)	19
Total	57 (47.1)	31 (25.6)	12 (9.9)	8 (6.6)	13 (10.7)	121

Technical success ranged from 75 to 97.6%, with a total of 93.4%. Diagnostic success ranged from 87.5 to 100%, with a total of 93.8%. Subsequent therapeutic success ranged from 85.7 to 100%, with a total of 96.2%. The overall success rate of the procedure was 84.3%. ERCP failure occurred in 19 patients, including failure to reach the target site in 8 patients (42.1%), failure of selective bile duct cannulation in 7 patients (36.8%), failure of stone removal in 1 patient (5.3%), and failure of stent insertion in 3 patients (15.8%). Details about the clinical outcomes were shown in Table 3.

**Table 3:** Summary of clinical outcomes

	Reaching the papilla or surgical anastomoses, %(n/N)	Diagnostic success, %(n/N)	Therapeutic success, %(n/N)	Overall success, %(n/N)
Billroth-I gastrectomy	87.5 (7/8)	100 (7/7)	85.7 (6/7)	75 (6/8)
Billroth-II gastrectomy	97.6 (81/83)	93.8 (76/81)	97.4 (74/76)	89.2 (74/83)
Roux-en-Y gastrectomy	75 (3/4)	100 (3/3)	100 (3/3)	75 (3/4)
Hepaticojejunostomy with Roux-en-Y	85.7 (6/7)	100 (6/6)	100 (6/6)	85.7 (6/7)
Pancreaticoduodenectomy	84.2 (16/19)	87.5 (14/16)	92.9 (13/14)	68.4 (13/19)
Total	93.4 (113/121)	93.8 (106/113)	96.2 (102/106)	84.3 (102/121)

### ERCP-related adverse events

In general, ERCP-related adverse events occurred in 31 cases, accounting for 25.6% (31/121) of all the cases (Table 4). Hyperamylasemia occurred in 14 cases (11.6%), and pancreatitis occurred in 11 cases (9.1%). Bleeding, mucosal laceration and perforation respectively occurred in 3 cases (2.5%), 2 cases (1.7%) and 1 case (0.8%). Of the 3 bleeding cases, 2 patients were dealt with hemostatic clips at once, and 1 patient with delayed bleeding underwent emergency endoscopic hemostasis using balloon compression and hemostatic clips. Of the 2 mucosal laceration cases, titanium clips were used to clamp the mucosal injury. The only perforation occurred in the acute angulation of the afferent loop. It was treated with purse string suture endoscopically. Hyperamylasemia and pancreatitis cases were treated conservatively. All the patients received complete recovery ultimately without surgical intervention.

**Table 4:** Outcomes of ERCP-related adverse events

	Pancreatitis	Hyperamylasemia	Mucosal laceration	Cholangitis	Bleeding	Perforation	Total, n/N (%)
Total, n (%)	11 (9.1)	14 (11.6)	2 (1.7)	0	3 (2.5)	1 (0.8)	31/121 (25.6)
Billroth-I gastrectomy	1	1	0	0	0	0	2/8 (25.0)
Billroth-II gastrectomy	9	11	0	0	3	0	23/83 (27.7)
Roux-en-Y gastrectomy	0	1	0	0	0	0	1/4 (25.0)
Hepaticojejunostomy with Roux-en-Y	1	0	1	0	0	0	2/7 (28.6)
Pancreaticoduodenectomy	0	1	1	0	0	1	3/19 (15.8)

### Risk factors for ERCP-related adverse events

In univariate analysis, not first time ERCP attempt (*p* = 0.121), a CBD stone diameter ≥ 15 mm (*p* = 0.191), multiple cannulation attempts (*p* = 0.077), endoscopic papillary balloon dilation (*p* = 0.036), endoscopic papillary large balloon dilation (*p* = 0.183), endoscopic retrograde biliary drainage (*p* = 0.125), biopsy in the bile duct or papilla (*p* = 0.055), mechanical lithotripsy use (*p* = 0.036), and stone retrieval basket (*p* = 0.163) were associated with ERCP-related adverse events (Table 5). In multivariate analysis, multiple cannulation attempts (OR 5.283; 95% CI 1.088–25.659; *p* = 0.039), endoscopic papillary balloon dilation (OR 4.381; 95% CI 1.191–16.114; *p* = 0.026), and biopsy in the bile duct or papilla (OR 35.432; 95% CI 2.693–466.104; *p* = 0.007) were independently associated with ERCP-related adverse events (Table 6).

**Table 5:** Factors of adverse events: univariate analysis

Variable	Adverse event (+) (n = 31), n (%)	Adverse event (−) (n = 90), n (%)	OR (95% CI)	*P* value
Age	–	–	0.996 (0.964, 1.028)	0.790
Male gender	25	64	1.693 (0.622, 4.605)	0.303
Prior cholecystectomy	11	29	1.157 (0.490, 2.729)	0.739
Roux-en-Y reconstruction	3	8	1.098 (0.272, 4.429)	0.895
Malignant biliary obstruction	7	16	1.349 (0.496, 3.668)	0.558
Without CBD stones	12	35	0.992 (0.429, 2.294)	0.986
Not first time ERCP attempt	6	31	0.457 (0.169, 1.231)	0.121
CBD stone diameter ≥ 15 mm	4	5	2.519 (0.631, 10.053)	0.191
Cannulation with difficulty or failure	6	12	1.560 (0.531, 4.587)	0.419
Multiple cannulation attempts	5	5	3.269 (0.878, 12.178)	0.077
Pancreatic deep wire pass	4	9	1.333 (0.380, 4.681)	0.653
Endoscopic sphincterotomy	2	7	0.818 (0.161, 4.163)	0.809
Needle-knife precut	2	6	0.966 (0.184, 5.053)	0.967
Endoscopic papillary balloon dilation	22	44	2.556 (1.061, 6.154)	0.036
Endoscopic papillary large balloon dilation (diameter > 10 mm)	13	26	1.778 (0.762, 4.145)	0.183
Endoscopic nasobiliary drainage	18	46	1.324 (0.581, 3.021)	0.504
Endoscopic retrograde biliary drainage	6	8	2.460 (0.779, 7.764)	0.125
Endoscopic retrograde pancreatic drainage	1	6	0.467 (0.054, 4.037)	0.489
Endoscopic metal biliary endoprosthesis	5	12	1.250 (0.402, 3.884)	0.700
Biopsy in the bile duct or papilla	3	1	9.536 (0.953, 95.366)	0.055
Mechanical lithotripsy use	4	2	6.519 (1.131, 37.560)	0.036
Stone balloon catheter	17	39	1.588 (0.699, 3.609)	0.270
Stone retrieval basket	19	42	1.810 (0.787, 4.162)	0.163
Intraductal-ultra sonography	2	3	2.000 (0.318, 12.567)	0.460
Presence of diverticulum	5	14	1.044 (0.343, 3.180)	0.940
Presence of a native papilla	26	71	1.392 (0.471, 4.109)	0.550
With a transparent hood	22	56	1.484 (0.613, 3.596)	0.382

OR: Odds ratio; CI: confidence interval

**Table 6:** Factors of procedural failure: multivariate analysis

Variable	OR (95% CI)	*P* value
Multiple cannulation attempts	5.283 (1.088, 25.659)	0.039
Endoscopic papillary balloon dilation	4.381 (1.191, 16.114)	0.026
Biopsy in the bile duct or papilla	35.432 (2.693, 466.104)	0.007

OR: Odds ratio; CI: confidence interval

## Discussion

With the development of endoscopic ultrasound (EUS) examination and magnetic resonance cholangiopancreatography (MRCP), ERCP has evolved into a therapeutic tool gradually. However, ERCP is challenging in patients with surgically altered anatomy. No standardized guidelines have been provided to choose the safest and most effective approach. Therefore, we retrospectively analyzed the outcomes, adverse events and related risk factors of 121 cases in 93 patients with surgically altered anatomy in our center.

ERCP procedure faces 3 challenges for these patients: reaching the target site, selective biliary cannulation, and performing diagnostic or therapeutic interventions [8]. The success rate of ERCP in patients with a reconstructive gastrointestinal tract has been reported to be 63–95% [30]. In our study, success rate of reaching the papilla or surgical anastomosis was 93.4%. Successful selective cannulation and cholangiography were achieved in 93.8% of them, which is similar to the rate of the same procedure using double-balloon enteroscopes (94%) [5]. In general, success rate of ERCP procedure in patients with surgically altered anatomy in our center was superior.

Success rates have increased with the introduction of device-assisted enteroscopes (DAEs), such as double-balloon enteroscopes, single-balloon enteroscopes and spiral enteroscopes. A previous review has reported an overall 74% ERCP success using DAEs [14]. A retrospective study by Shah et al. has shown a 63% ERCP success with DAEs [12]. Inamdar et al. reported a pooled technical, diagnostic, and procedural success rates of 80.9%, 69.4%, and 61.7% respectively with single-balloon enteroscopes [31]. Corresponding success rates were reported to be 86%, 100%, and 86% by Ali et al. with spiral enteroscopes in Roux-en-Y anatomy [20]. Newly designed features in DAEs, such as high force transmission and passive bending, made it possible to increase the success rates of ERCP procedures in these patients further [32, 33]. However, DAEs are not designed for this purpose and have not been widely available because of the need for specialized equipment and expertise. In our study, standard enteroscopes were proved effective and safe to perform in patients with surgically altered anatomy, which brought better applicability.

Despite emerging enteroscopes applied in patients with surgically altered anatomy, ERCP-related success rates are still less satisfactory than in patients with normal gastrointestinal anatomy. The procedure is considered challenging for several reasons. First, an increase of the intestine length, and sharp angulation of the bowel make it difficult to identify the afferent loop and reach the target site [10]. Existing enteroscopes tend to be too short to approach the papilla or anastomosis. Second, selective cannulation is difficult due to the unfavorable orientation of the papilla, along with limited availability of accessories, and lack of an elevator [34]. Third, adhesions and strictures left by reconstruction also impede the process. Techniques such as manual compression method, positional change, and use of a transparent cap have been proposed to increase the success rates [35].

Since no standardized procedure has been applied in these patients, a thorough understanding of postoperative anatomy and multiple training seem extremely important. Relative knowledge of the reconstruction method, biliary anastomosis, the lengths of the limbs, and the presence of adhesions and strictures is indispensable. In addition, proper selection of endoscopes is likely to determine the outcome of the ERCP procedure. Selection of endoscopes was mainly depended on patients’ anatomy of the gastrointestinal tract, such as lengths of afferent and efferent loops, degrees of angulations, and endoscopists’ operating experience. In our study, technical success was highest in patients with Billroth-II gastrectomy, while lowest in patients with Roux-en-Y gastrectomy. However, ERCP in patients with Roux-en-Y reconstruction had perfect diagnostic success and therapeutic success. Longer afferent limbs, more severe adhesion in Roux-en-Y reconstruction make it particularly difficult to reach the blind end. Therefore, important drawbacks appeared when using a conventional side-view duodenoscope in these patients. A short-type single-balloon enteroscope may be useful to address this issue. With a passive bending part, it contributed to the advancement of the scope. The balloon also helps to hold and fix the intestine, making it possible to insert deeply. Considering an increase of Roux-en-Y reconstruction due to the growing need for laparoscopic surgeries [36], special attention is required to this group. Conventional duodenoscopes and gastroscopes are recommended to patients with Billroth-I or Billroth-II gastrectomy, owing to the relatively short afferent limbs. As for Billroth-II reconstruction, it is under debate whether to choose a forward-view or a side-view enteroscope [11]. In our study, several patients with Billroth-II reconstruction changed the enteroscope midway, from a gastroscope to a duodenoscope. A forward-view gastroscope can provide better visualization, making it safer to reach the papilla. Then with a side-view duodenoscope, cannulation becomes easier due to the appropriate view to the papilla. Similar results have been raised by Park et al. The forward-view endoscope was associated with a higher afferent loop intubation rate, while the side-view endoscope with a higher selective cannulation rate [37]. Enteroscope exchange strategy is expected to be more effective for these patients.

In our present study, ERCP-related adverse events occurred in 25.6% of patients. Previous studies reported the complication rate from 3.5 to 12.4% [9, 11, 12, 16, 18, 38–40]. However, hyperamylasemia was not included in most of these studies. A comparable 23.0% ERCP-related complication rate was reported combined with hyperamylasemia [35]. Post-ERCP pancreatitis (PEP) is recognized as the most common procedure-related adverse event in conventional ERCP, while perforation in balloon-assisted ERCP [7]. A systematic review based on randomized controlled trials reported an overall 9.7% rate of PEP [41]. Other complications include bleeding, cholangitis, mucosal laceration and cardiopulmonary related diseases. Recently, the advent of DAEs has allowed lower rates of ERCP-related adverse events. It was reported in patients with Roux-en-Y reconstruction using DAEs with complication risks ranging from 0 to 19.5% [42]. A meta-analysis concluded an overall 6.5% complication rate in 461 patients using single-balloon enteroscopes [31]. Anvari et al. reported a 4% complication rate in 1523 patients with double-balloon enteroscopes [5]. However, DAEs are more technically demanded and bring a higher risk of complications. Conventional ERCP allows more practical use. In our center, no lethal complication occurred, with most adverse events treated conservatively. Conventional enteroscopes were equally effective and safe.

Our study showed that multiple cannulation attempts, endoscopic papillary balloon dilation, and biopsy in the bile duct or papilla were independent risk factors of ERCP-related adverse events. Multiple cannulation attempts mean prolonged procedure time and usually follow a pre-cut sphincterotomy, which increases the risk of PEP and bleeding. An increased rate of PEP has been seen with additional cannulation attempts in a prospective study [43]. In our study, higher rates of PEP and hyperamylasemia were likely to be associated with EPBD, which concurred with the study reported by Park et al. [35]. Previous studies have reported that compared with endoscopic sphincterotomy (EST) or endoscopic sphincterotomy with balloon dilatation (ESBD), EPBD presented higher rates of pancreatitis, while bleeding was more common in the former factors [44, 45]. The reason may due to mucosal edema of the papillary caused by procedures, leading to pancreatic outflow obstruction. In addition, biopsy in the bile duct or papilla can also increase the complication rate.

To our knowledge, few studies have been conducted to identify the risk factors of ERCP-related adverse events including multiple types of reconstruction methods and endoscopes. Compared with previous studies, our present study had a relatively larger number of patients, a more comprehensive analysis of all the reconstruction methods and endoscopes, making it more practical and applicable. However, several limitations did exist, including its retrospective design based on a single-center experience, lack of follow-up data and a control group, variations of patients, reconstruction methods and endoscopes. In the future, multi-center prospective studies are needed to validate present findings. Establishment of standardized practical guidelines and training programs are indispensable to guide ERCP procedures in patients with surgically altered anatomy.

In conclusion, ERCP procedure was feasible and safe in patients with surgically altered anatomy. For early identification of the occurrence of ERCP-related adverse events, close vigil should be kept on patients who have undergone multiple cannulation attempts, endoscopic papillary balloon dilation, and biopsy in the bile duct or papilla.

## Data Availability

The datasets supporting the conclusions of this article are included within the article. Correspondence to Qiu Zhao if necessary.

## Abbreviations

ERCP: Endoscopic retrograde cholangiopancreatography
DAEs: Device-assisted enteroscopes
CBD: Common bile duct
EPBD: Endoscopic papillary balloon dilation
ORs: Odds ratios
CIs: Confidence intervals
SBE: Single-balloon enteroscope
EUS: Endoscopic ultrasound
MRCP: Magnetic resonance cholangiopancreatography
PEP: Post-ERCP pancreatitis
EST: Endoscopic sphincterotomy
ESBD: Endoscopic sphincterotomy with balloon dilatation
